# Intraosseous Ganglia: A Series of 17 Treated Cases

**DOI:** 10.1155/2013/462730

**Published:** 2013-06-13

**Authors:** Akio Sakamoto, Yoshinao Oda, Yukihide Iwamoto

**Affiliations:** ^1^Department of Orthopaedic Surgery, Graduate School of Medical Sciences, Kyushu University, 3-1-1 Maidashi, Fukuoka 812-8582, Japan; ^2^Department of Anatomic Pathology, Graduate School of Medical Sciences, Kyushu University, Fukuoka 812-8582, Japan

## Abstract

*Background*. Intraosseous ganglion is a cystic lesion that contains gelatinous material, most often occurs in middle-aged patients, and is regarded as similar to soft-tissue ganglion. The etiology is unknown, but association with degenerative joint disease has been considered. *Materials and Methods*. At a single institute, 17 patients (8 men, 9 women) with a mean age of 48.9 years (22–72 years) were surgically treated for an intraosseous ganglion. The lesions were located in 9 long bones (5 tibiae, 2 humeri, 1 ulna, and 1 femur); 4 flat bones (2 scapulae, 2 ilia); and 4 small bones (2 scaphoid, 1 metacarpal bone, and 1 talus). The diagnosis was confirmed based both on the gross intraoperative finding of intralesional gelatinous material and on histopathology. *Results*. All lesions occurred at the epiphysis or near the joint. The plain radiographs showed a lesion with marginal osteosclerosis. The average lesion size was 22.4 mm (range 6–40 mm). Among the 17 patients, 2 (12%) had osteoarthritis, 3 (18%) had pathological fracture, and 4 (24%) had extraskeletal extension. *Discussion and Conclusion*. The periosteum and cortex of bone represent physical barriers. Therefore, it seems much more likely that primary bone lesions will spread to the soft tissues. Intraosseous ganglion does not appear to be associated with either soft-tissue ganglion or with osteoarthritis. This clinical information and the appearance on plain radiographs, particularly the marginal osteosclerosis, are of differential diagnostic importance.

## 1. Introduction

Intraosseous ganglion is a benign, nonneoplastic bone lesion with histological similarity to that in soft tissue [1–3]. Intraosseous ganglion contains mucoid viscous material with no epithelial or synovial lining [4]. Peak incidence of intraosseous ganglion is in the 4th and 5th decades of life, and it is rare in children [5, 6]. Most reports of intraosseous ganglion in the biomedical literature describe a single case or a series of a few cases. In the current retrospective medical record study, the clinical features of intraosseous ganglion in 17 patients treated at one institute were assessed. 

## 2. Materials and Methods

**Figure 1:**
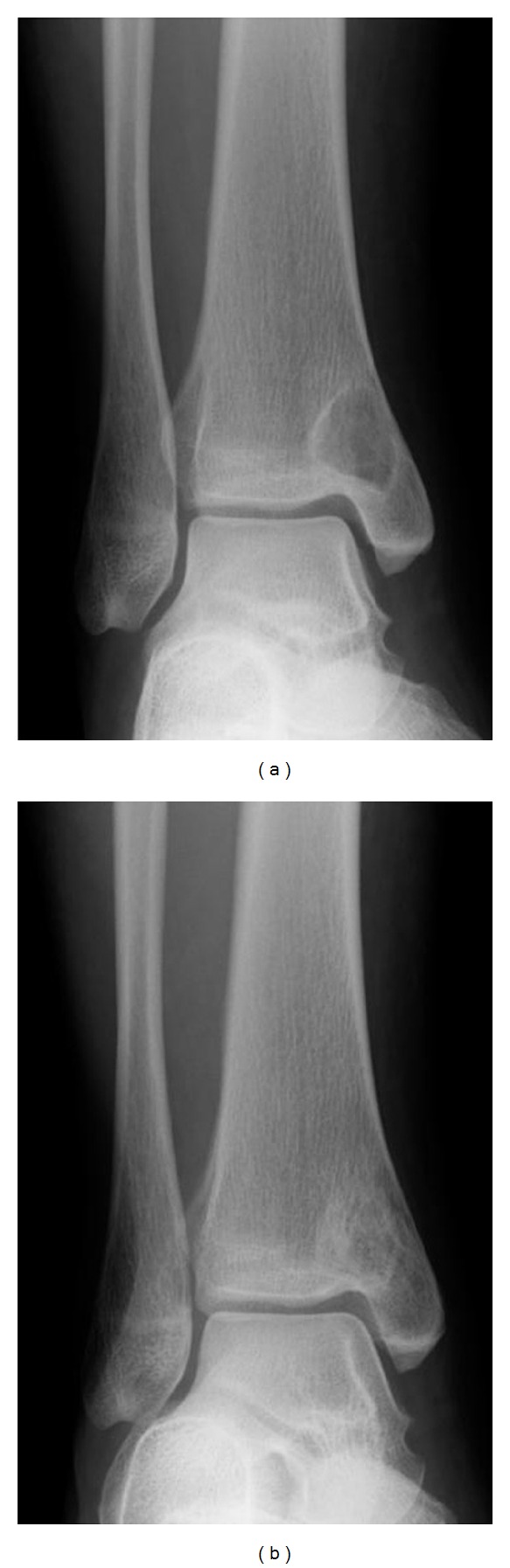
Intraosseous ganglion of the distal tibia in a 37-year-old woman. Plain radiograph shows an osteolytic lesion with marginal osteosclerosis. Three years after the operation of curettage and bone grafting, osteosclerosis is seen.

A retrospective medical record review showed that 17 patients (8 men, 9 women) with a final diagnosis of an intraosseous ganglion were seen at our institute during the 6 years from 2004 to 2009. All cases were introduced, and during this time, all suspected intraosseous ganglion lesions were treated as an extension of biopsy. The initial diagnosis of intraosseous ganglion was made based on the plain radiographs. Intraosseous ganglia appeared as well-circumscribed radiolucent lesions accompanied by marginal sclerosis (Figure 1). Curettage was performed in all cases except one, which was treated using arthroplasty. Bone grafting was performed in all the curetted lesions except for one metacarpal lesion. The diagnosis of intraosseous ganglion was confirmed by the gross intraoperative finding of jelly-like material within the lesion and by histopathology. Patient age and lesion size were statistically analyzed using the Mann-Whitney *U* test. A *P* value of less than 0.05 was considered to indicate statistical significance. Each patient and their family members were informed that the data from their case would be submitted for publication, and their consent was obtained.

## 3. Results

Clinical data are summarized in Tables 1 and 2. Of the 17 lesions, 9 were in long bones, 4 in flat bones, and 4 in small bones of the hand and feet. Among the 9 long bone lesions, 3 were proximal and 6 were distal. Among the 17 cases, 6 (40%) were in weight-bearing long bones of the lower limb.

In the 9 long-bone lesions, right predominance was seen (7 right, 2 left). However, neither side predominated in the flat bone and small bone lesions.

The average patient age overall was 48.9 years (range 22–72); 2 patients were younger than 30 years, and 2 were between 30 and 40 years old. The average age of patients with an intraosseous ganglion in the long bone was 51.4 years, which was not significantly different from that of patients with an intraosseous ganglion in a flat bone (47.3 years) or in a small bone (45.0 years). The average size of an intraosseous ganglion overall was 22.4 mm (range, 6–40 mm); in long bones was 23.7 mm; in flat bones was 31.3 mm; and in small bones was 10.8 mm. No statistically significant difference was found in the size of lesions by bone type. Fracture was present in 3 of the 17 cases (18%): one in the scaphoid bone and 2 in the ilium, the latter comprising 2 of the 2 ilia (100%). Osteoarthritis was seen in 2 cases (13%) and soft-tissue extension was seen in 4 cases (24%). Continuity to the nearby joint was not observed in all cases. Bone consolidation within 1 year after surgery occurred in all 16 cases that had been treated with curettage. No recurrence was observed in any case after at least 3 years of followup.

## 4. Discussion

Nonoperative observation is an option for intraosseous ganglia [1]. In the current study, because almost all cases of an intraosseous ganglion were treated surgically, the clinical data regarding the anatomical site and ages appear to be accurate. The overall average age of the current series of patients was 48.9 years, a result consistent with the reported age at diagnosis of intraosseous ganglia [5]. However, it should be noted that in the current series 2 patients were less than 30 years old, and 2 patients were between 30 and 40 years old. The prevalence of intraosseous ganglia has been reported to have a small male preponderance [7], but no significant difference based on sex was seen in the current series. The most common bone affected in this study was the tibia, a result consistent with that of previous studies showing a tendency for the long bones of the lower limb; however, the carpal bones are another well-recognized site [5, 8–11].

On plain radiographs, the intraosseous ganglion appears as a well-defined osteolytic lesion located near a joint. Most intraosseous ganglia are small, between 1 and 2 cm in maximum diameter; lesions over 5 cm are rare [4, 5]. The differential diagnoses include tumors that arise in the epiphyseal to metaphyseal region, such as giant cell tumor of bone, aneurysmal bone cyst, and chondroblastoma [4, 5, 9]. Giant cell tumor of bone and aneurysmal bone cyst are typically large and can be differentiated from intraosseous ganglion on radiographs by a lack of marginal osteosclerosis and thinning of the adjacent cortex due to expansion. Differentiation from chondroblastoma can be difficult, as the lesion typically has marginal osteosclerosis. However, chondroblastomas tend to occur in younger patients. Confirmation of the cystic nature of the lesion by gadolinium enhancement may be helpful for diagnosis of an intraosseous ganglion by lack of enhancement.

Intraosseous ganglion is a cystic lesion that contains gelatinous material and is regarded as similar to that of soft-tissue ganglion. Synovial fluid intrusion is the currently favored pathological mechanism of soft-tissue ganglion [12]. The etiology of intraosseous ganglion is unknown. One theory is that intraosseous ganglion may be caused by penetration of a soft-tissue ganglion into the underlying bone [1, 10]. However, the reported frequency of extraosseous extension is only 16% [5] and was 24% in the current series. Moreover, the periosteum and cortex of bone represent substantial physical barriers to intraosseous extension of a soft-tissue lesion into the bone. Therefore, it seems much more likely that primary bone lesions spread to the soft tissues.

 In the current series, osteoarthritis was seen in 12% of patients, while 16% of intraosseous ganglia are reported to be associated with degenerative joint disease [6]. There is less association between intraosseous ganglia and degenerative joint disease than previously reported. An intraosseous ganglion is considered to be a lesion that is distinct from a degenerative subchondral cyst [4]. The pathogenesis of degenerative subchondral cysts is suggested to be synovial fluid intrusion [4]. Intraosseous ganglia rarely communicate with a joint cavity [5]. In the current case series, an obvious continuity to the nearby joint was not observed in many cases. However, one hypothesis suggests that the passage of synovial fluid causes an intraosseous ganglion through a small defect of cartilage and subchondral bone [2]. The possible fine communication from a nearby joint to an intraosseous ganglion has been consistently reported after arthroscopy [2] and an arthrographic procedure [3]. This type of communication has also been seen in imaging of an intraosseous ganglion, which was presumably of articular origin [3].

 In conclusion, plain radiographs as well as clinical information are important for the accurate diagnosis of intraosseous ganglion. The pathogenesis is unknown, but intraosseous ganglion does not appear to be associated with either soft-tissue ganglion or with osteoarthritis.

## Figures and Tables

**Table 1:** Clinical features of cases of intraosseous ganglion.

Case	Age/sex	Side/location	Proximal/distal	Epiphysis (nearby joint)	Osteoarthritis	Size	Marginal sclerosis	Fracture	Soft-tissue extension	Bone graft
G1	70/F	R/tibia	Proximal	+	+	23 mm	+	−	+	+ (auto)
G2	42/M	L/scaphoid	Distal	+	−	6 mm	+	+ (pseudojoint)	−	+ (auto)
G3	47/F	R/scapula	Distal	+	−	25 mm	+	−	+	+ (auto)
G4	36/F	R/ulna	Distal	+	−	20 mm	+	−	−	+ (auto)
G5	37/F	R/tibia	Distal	+	−	20 mm	+	−	−	+ (auto)
G6	69/M	L/tibia	Proximal	+	−	35 mm	+	−	−	+ (auto)
G7	44/F	L/ilium	Distal	+	−	30 mm	+	+	−	+ (auto)
G8	51/M	R/scapula	Distal	+	−	30 mm	+	−	−	+ (auto)
G9	22/F	R/metacarpal	Proximal	+	−	7 mm	+	−	−	−
G10	54/F	R/scaphoid	Distal	+	−	10 mm	+	−	−	+ (auto)
G11	62/F	L/talus	Proximal	+	−	20 mm	+	−	−	+ (auto)
G12	72/M	L/humerus	Distal	+	+	40 mm	+	−	+	+ (auto)
G13	49/M	R/tibia	Proximal	+	−	30 mm	+	−	+	+ (auto)
G14	47/F	L/ilium	Distal	+	+	40 mm	+	+	−	+ (auto + alo)
G15	24/M	R/humerus	Distal	+	−	10 mm	+	−	−	+ (auto)
G16	40/F	R/tibia	Distal	+	−	12 mm	+	−	−	+ (auto)
G17	66/M	R/femur	Distal	+	−	23 mm	+	−	−	−

M: male; F: female; L: left; R: right; auto: autograft; alo: allograft.

**Table 2:** Clinical summary of intraosseous ganglion.

	Age	Sex (male/female)	Side (right/left)	Osteoarthritis	Size (mm)	Fracture	Soft-tissue extension
Large long bone (*n* = 9) Tibia (*n* = 5: proximal 3, distal 2) Humerus (*n* = 2: distal 2) Ulna (*n* = 1: distal) Femur (*n* = 1: distal)	51.4 (24–72)	4/5	7/2	2/9 (22%)	23.7 (10–40)	0/9 (0%)	3/9 (33%)

Flat bone (*n* = 4) Scapula (*n* = 2) Ilium (*n* = 2)	47.3 (44–51)	2/2	2/2	0/4 (0%)	31.3 (25–40)	2/4 (50%)	1/4 (25%)

Bones in the hand and foot (*n* = 4) Scaphoid (*n* = 2) Metacarpal bone (*n* = 1) Talus (*n* = 1)	45.0 (22–62)	2/2	2/2	0/4 (0%)	10.8 (6–20)	1/4 (25%)	0/4 (0%)

Total (*n* = 17)	48.9 (22–72)	8/9	11/6	2/17 (12%)	22.4 (6–40)	3/17 (18%)	4/17 (24%)
